# iPSC-derived neuronal models of PANK2-associated neurodegeneration reveal mitochondrial dysfunction contributing to early disease

**DOI:** 10.1371/journal.pone.0184104

**Published:** 2017-09-01

**Authors:** Charles Arber, Plamena R. Angelova, Sarah Wiethoff, Yugo Tsuchiya, Francesca Mazzacuva, Elisavet Preza, Kailash P. Bhatia, Kevin Mills, Ivan Gout, Andrey Y. Abramov, John Hardy, James A. Duce, Henry Houlden, Selina Wray

**Affiliations:** 1 Department of Molecular Neuroscience, Institute of Neurology, University College London, London, United Kingdom; 2 Institute of Structural and Molecular Biology, University College London, London, United Kingdom; 3 Centre for Translational Omics, Genetics and Genomic Medicine Programme, UCL Institute of Child Health, London, United Kingdom; 4 School of Molecular and Cellular Biology, Faculty of Biological Sciences, University of Leeds, Leeds, United Kingdom; CINVESTAV-IPN, MEXICO

## Abstract

Mutations in *PANK2* lead to neurodegeneration with brain iron accumulation. PANK2 has a role in the biosynthesis of coenzyme A (CoA) from dietary vitamin B5, but the neuropathological mechanism and reasons for iron accumulation remain unknown. In this study, atypical patient-derived fibroblasts were reprogrammed into induced pluripotent stem cells (iPSCs) and subsequently differentiated into cortical neuronal cells for studying disease mechanisms in human neurons. We observed no changes in *PANK2* expression between control and patient cells, but a reduction in protein levels was apparent in patient cells. CoA homeostasis and cellular iron handling were normal, mitochondrial function was affected; displaying activated NADH-related and inhibited FADH-related respiration, resulting in increased mitochondrial membrane potential. This led to increased reactive oxygen species generation and lipid peroxidation in patient-derived neurons. These data suggest that mitochondrial deficiency is an early feature of the disease process and can be explained by altered NADH/FADH substrate supply to oxidative phosphorylation. Intriguingly, iron chelation appeared to exacerbate the mitochondrial phenotype in both control and patient neuronal cells. This raises caution for the use iron chelation therapy in general when iron accumulation is absent.

## Introduction

Neurodegeneration with brain iron accumulation (NBIA) disorders are a set of clinically analogous neurological diseases characterised by neuropathology of the basal ganglia coinciding with iron deposition [1]. Patients display pyramidal and extrapyramidal movement disruption as well as cognitive decline. Pathological examination highlights either axonal swellings with ubiquitinated aggregates, tau tangles or Lewy bodies depending on the NBIA subtype. Mutations in 12 genes have been shown to cause NBIA and each protein has a seemingly disparate cellular function [2]. These functions include iron metabolism, mitochondrial metabolism, lipid homeostasis and autophagy.

The most common NBIA subtype is pantothenate kinase-associated neurodegeneration (PKAN), caused by recessive mutations in the *PANK2* gene [3]. This accounts for 35–50% of all NBIA cases [4,5]. Pantothenate kinase (PANK) catalyses the first step of coenzyme A (CoA) biosynthesis from dietary vitamin B5. CoA has critical roles in multiple mitochondrial metabolic pathways, including the TCA cycle, β-oxidation and fatty acid synthesis. There are four human PANK isoforms; PANK1 and PANK3 are cytosolic, whereas PANK2 is localised to the mitochondria [6]. There is still some contention over the localisation of mouse Pank2 between the mitochondrial membranes [7] and the cytosol [6,8]. PANK4 is an isoform presumed to lack catalytic activity [9]. CoA is present in the mitochondrial matrix at 1000-fold higher levels than in the cytosol [10] and PANK2 is the major active PANK isoform in the human brain. Despite rodent brain tissue being less enriched for Pank2 than human brain, its central role is demonstrated as *Pank2* knockout mice have 60% reduced total PANK activity in neural tissue [11]. These data support a primary role for PANK2 and CoA in neuronal mitochondria. However, mitochondrial CoA is yet to be measured in patient-derived or mouse model brain tissue.

The mechanism by which *PANK2* mutations lead to neurodegeneration is not known but several animal models have been generated to facilitate investigation of disease mechanisms. *Drosophila* have one Pank orthologue and deletion partially recapitulates some of the movement phenotypes and reduced lifespan observed in PKAN [12,13]. Addition of human mitochondrial PANK2 is able to rescue this phenotype [13]. Interestingly, while *Drosophila* cytosolic Pank isoforms are not able to rescue knockout fly phenotypes, addition of the human cytosolic isoforms provide a partial rescue. *Pank2* knockout mice also show a similar phenotype, but only when metabolically stressed with a ketogenic diet [14]. These animal models fail to display iron accumulation. Patient fibroblasts have been shown to display defective iron handling, increased reactive oxygen species damage (ROS) and mitochondrial physiological deficits [15]–findings that were replicated in human neurons for the first time after direct reprogramming from patient fibroblasts [16] and subsequently reinforced in iPSC-derived neurons [17].

PKAN patients display iron accumulation in the globus pallidus and have cellular pathology, namely axonal swellings and gliosis, affecting the cortex as well as the neurons of the globus pallidus [18,19]. Despite being an essential element of cell survival, it is unclear whether iron accumulation is causative or consequential to neurodegeneration. Many cellular enzymes make use of heme iron for normal folding and function as well as iron-sulphur clusters for enzymatic function; notable examples are the complexes of the electron transport chain of oxidative phosphorylation. Deregulated iron can be potentially harmful to the cell as, depending on its oxidative state, it can lead to free radical formation via the Fenton reaction. Therefore, tight cellular mechanisms for import, storage and export of iron from the cell exist [20]. Neuronal iron is predominantly imported via endocytosis of the Transferrin Receptor (TfR) and either stored intracellularly through complexes such as Ferritin, consisting of both heavy (FTH) and light (FTL) chains, or utilized immediately by the cell for normal function. For its use in aerobic respiration, iron is imported into the mitochondria via mitoferrin transporters (MFRN1/2) and mitochondrial specific ferritin (MTFT) stores mitochondrial iron until it is required or when in excess. Iron is exported from neurons via Ferroportin (FPN), which requires cell surface stabilization through binding to β-amyloid precursor protein [21,22]. Iron within a healthy cell is predominantly contained within mitochondria and lysosomes [23] and upon entering the mitochondria it is thought to accumulate as no mitochondrial iron exporter has been described thus far. This has led to the suggestion that mitophagy may be a mechanism for liberating iron from mitochondrial stores [24].

The present study sets out to investigate the consequences of *PANK2* mutations on iPSC-derived cortical neuronal cells in culture. Fibroblasts from three atypical PKAN patients were reprogrammed to iPSCs and, along with three control pluripotent stem cell (PSC) lines, differentiated into cortical neuronal cells using a highly efficient differentiation paradigm. Mitochondrial dysfunction was observed, namely altered NADH and FADH supply to oxidative phosphorylation as well as increased reactive oxygen species (ROS) production and oxidative damage. Changes to iron and CoA metabolism were not witnessed. Additionally, it was shown that iron chelation led to increased oxidative damage in patient and control neuronal cells. These findings enable analysis of early pathological events in PKAN without the context of aging and complex late-stage disease.

## Materials and methods

### Cell culture and reprogramming of fibroblasts

All culture reagents were purchased from Thermo Fisher unless otherwise stated. Patient biopsies were taken using a skin punch under informed consent (ethical approval from the NHNN and IoN joint research ethics committee, study number 10/H0721/87). Fibroblasts were cultured as previously described [25]. Briefly, 5mm biopsies were taken with a skin punch and then allowed to expand in DMEM supplemented with 10% FBS. Fibroblasts were passaged using 0.05% trypsin/EDTA.

Fibroblasts were reprogrammed using the episomal plasmids as described by Okita et. al. [26]. The episomal plasmids were obtained from Addgene (plasmids #27077, #27078 and #27080) and fibroblasts were nucleofected using the Lonza P2 nucleofection kit (Amaxa). Nucleofected cells were changed to iPSC culture media after 7 days and colonies were manually picked after they appeared around 30 days post nucleofection. iPSCs and hESCs were maintained in Essential 8 media on Geltrex coated plates. Cells were routinely passaged using 0.5 mM EDTA. Karyotype counts and G banding analysis was performed by Cell Guidance Systems (Cambridge, UK).

At least two clonal iPSC lines from each patient were taken forward for experimentation and compared to two control iPSC lines and one hESC line; termed Control 1, Control 2 and hESC Control respectively. The hESC line Shef6 was obtained from the UK Stem Cell Bank, Control iPSC line 1 was generated from a neurologically normal individual in the lab of Dr Tilo Kunath and Control iPSC line 2 was obtained from the Coriell repository.

Stem cells were differentiated to cortical neuronal cells using the protocol described by Shi et. al. [27]. Briefly, cells were subjected to 10 days dual SMAD inhibition using 1 μM dorsomorphin (Tocris) and 10 μM SB431542 (Tocris), followed by extended neurogenesis in N2B27 media containing retinoids. The final time point for all experiments was taken as 100 days post neural induction.

### Immunocytochemistry

Cells were fixed in 4% PFA, washed three times in PBS with 0.3% Triton X-100 (PBST) to permeabilize the cells and blocked in 3% BSA. Primary antibodies (Table 1) were incubated overnight at 4°C in blocking solution. Cells were then washed three times in PBST and secondary antibodies (Alexa Fluor, Thermo Fisher) were added for 1 hour at room temperature in the dark in blocking solution. Finally, cells were washed once with PBST containing 1 μM DAPI and then twice in PBST before being fixed and mounted. Images were taken on a Zeiss LSM microscope or a Zen confocal microscope. Counting data was taken from 5 images per replicate; areas were randomly selected in the DAPI channel and automated counting was performed using the ITCN nuclear counting plugin for Image J, using the same threshold setting throughout.

**Table 1:** Antibodies used for ICC and Western blotting.

Antibody Name	Source	Dilution	Species
**OCT4**	Santa Cruz sc-5297	1:500	gt
**SSEA4**	BioLegend MC-813-70	1:200	m
**OTX2**	Millipore AB9566	1:400	rb
**Ki67**	BD 550609	1:500	m
**TUJ1**	BioLegend 801201/802001	1:1000	m/rb
**TBR1**	Abcam ab31940	1:500	rb
**SATB2**	Abcam ab51502	1:100	m
**CTIP**	Abcam ab18465	1:500	rat
**PANK2 (3H9)**	Abcam ab119070	1:1000	m
**TfR**	Invitrogen 13–6800	1:500	m
**FTH1**	NEB 3998	1:500	rb
**GAPDH**	Ambion AM4300	1:5000	m

### qPCR

RNA was isolated from samples using Trizol reagent and purification was performed following the manufacturers’ instructions (Thermo Fisher). Reverse transcription was performed on 2 μg of RNA using Superscript reverse transcriptase III and random hexamer primers. Power Sybr Green mastermix (Thermo) was used for the qPCR reaction on the Agilent MX3000P qPCR system with annealing temperatures of 60°C for all primers used (Table 2). All results are relative to three housekeeping genes *GAPDH*, *Cyclophilin* and β*-actin*.

**Table 2:** Primers used for qPCR.

Gene	Forward Primer	Reverse Primer	Product
***GAPDH***	atgacatcaagaaggtggtg	cataccaggaaatgagcttg	177bp
***CYCLOPHILIN***	ggcaaatgctggaccaaacac	ttcctggacccaaaacgctc	147bp
***b-ACTIN***	tcaccaccacggccgagcg	tctccttctgcatcctgtcg	351bp
***OCT4***	Ttctggcgccggttacagaacca	gacaacaatgaaaatcttcaggaga	218bp
***SOX2***	catggcaatcaaaatgtcca	tttcacgtttgcaactgtcc	119bp
***NANOG***	Gcttgccttgctttgaagca	ttcttgactgggaccttgtc	256bp
***VIMENTIN***	gtacgtcagcaatatgaaag	agtgtcttggtagttagcag	270bp
***S100A4***	Ttctttcttggtttgatcc	ttagttctgacttgttgagc	211bp
***TfR***	gacgcgctagtgttcttc	actgttatcgccatctactt	136bp
***FPN***	tctctctacttggggagat	tcagaagctcatgtttatgta	266bp
***FTH***	cagaactaccaccaggact	agtatttggcaaagttcttc	136bp
***FTL***	tctcaagcacgactaagag	gcagaagccctattacttt	74bp
***MFRN1***	aatcgagctccatactaaag	gtcccctcctctctctaa	164bp
***MFRN2***	gtgatgtaccccatcgac	cgttcttataatcctccaga	108bp
***FTMT***	agtgtgctctactcttggaa	gtcacctttatctgaggcta	77bp
***PANK2***	tcagtcggattcaatgga	aagcagaggatacggatt	108bp

### Western blot analysis

Samples were treated with 50 μM FAC for 18 hours in normal cell culture media. Samples were lysed in RIPA buffer containing 10 mM Tris pH 8, 140 mM NaCl, 1 mM EDTA, 0.5 mM EGTA, 1% Triton X-100 0.1% sodium deoxycholate, 0.1% SDS plus protease and phosphatase inhibitors (Roche), for one hour on an orbital shaker at 4°C, followed by centrifugation at 10,000 g for 15 minutes at 4°C. Protein concentrations were measured using the BSA assay (Biorad) and samples were separated on a NuPage 10% SDS polyacrylamide gel (Novex) before being transferred onto a nitrocellulose membrane for western blotting. Membranes were blocked in 3% milk in PBS containing 0.1% Tween 20. Primary antibodies were added to the membranes in blocking solution overnight at 4°C. Blots were then washed three times before secondary antibodies were added in blocking solution for one hour. After final washes, images were captured and densitometry analysis performed on the Li-Cor Odyssey imaging system (Li-Cor).

### HPLC

CoA species were extracted from cells with ice-cold perchloric acid (PCA, 3.5%). After centrifugation at 21,000 g for 5 min at 4°C, the supernatant, containing CoASH and short-chain CoA esters, was collected and 1 M triethanolamine (TEA) was added to a final concentration of 100 mM. The pH was adjusted to pH 6 with 5 M K_2_CO_3_ and potassium perchlorate pellet was removed by centrifugation at 21,000 g for 3 minutes at 4°C. For the quantification of the total level of acid-soluble CoA esters (combined level of unesterified CoA and short chain CoA), 5 M KOH and 100 mM tris (2-carboxyethyl) phosphine were added to neutralized PCA extracts to final concentrations of 0.5 M and 10 mM, respectively. KOH hydrolyses all PCA-soluble esters into unesterified CoA which were then measured by HPLC. After incubation at 25° C for 5 minutes, the pH was adjusted to pH 6 with 5% PCA. CoASH and short-chain CoA esters were measured by HPLC as previously described [28] except EDTA was omitted from the injection mixture. For the quantification of total long-chain acyl CoAs, the PCA pellets were solubilised in 89 mM TEA, 0.44 mM KOH and 11.1 mM DTT by gentle sonication to hydrolyse long-chain CoA esters to unesterified CoA. After incubation for 5 min at 25° C, proteins were precipitated by PCA and pelleted by centrifugation at 21,000 g for 10 min at 4°C. The supernatant was collected and the pH was adjusted to 6–7 with 0.5 M K_2_CO_3_ and centrifuged again at 21,000 g for 3 min at 4°C. The supernatant was collected and CoA was measurement by the CoA recycling assay [29] adapted to a plate reader format.

### Mass spectrometry

The UPLC-MS/MS instrument consisted of a Waters ACQUITY UPLC system coupled to a Xevo TQ-S triple quadrupole mass spectrometer with an electrospray ionization source. The mass spectrometer was operated in negative ion mode and data were acquired using MassLynx V4.1 software. Chromatographic separations were achieved using a Waters CORTECS C_18_ column (1.6 μm, 2.1 x 50 mm), with a CORTECS C_18_ VanGuard pre-column (1.6 μm), which was maintained at 40°C. Binary gradient profiles were developed using water with 0.01% formic acid (A) and methanol (B) (HPLC grade, Merck) at a flow rate of 700 μL/min. Separations were conducted under the following chromatographic conditions: 100% solvent A for 1 min, decreased to 10% over 1 min, maintained for 1 min at 10% before being increased to 100% over 0.1 min. Column equilibration time was 0.9 min, with a total run time of 4 min. The injection volume was 10 μL. Mass spectrometric conditions were as follows: capillary voltage 2.5 kV, cone voltage 60 V, source temperature 150°C, desolvation temperature 600°C, cone gas flow 150 L/h, desolvation gas flow 800 L/h, collision gas flow 0.25 L/h and nebulizer gas flow 7 bar. Dwell time was set at 8 msec for each analyte. The quantitation of vitamin B5 and isotopically-labelled vitamin B5 (Sigma) was then performed using the multiple reaction monitoring (MRM) method described in Table 3. It is important to note for isotopically labeled pantothenate, treatment was performed in media deficient of pantothenate: HBSS media supplemented with N2 and B27 supplements, NEAA and L-glutamine as the above media recipes.

**Table 3:** MRM conditions for the analysis of vitamin B5 and isotopically-labelled vitamin B5 including retention times, collision energy, precursor ions and their respective quantifier and qualifier ions.

*Compound*	*Retention time (min)*	*Precursor ion* *(m/z)*	*Quantifier ion (m/z)*	*Collision energy quantifier (V)*	*Qualifier ion* *(m/z)*	*Collision energy* *qualifier (V)*
*vitamin B5*	*1*.*56*	*218*.*2*	*88*.*0*	*10*	*146*.*0*	*12*
^*13*^*C*_*6*_,^*15*^*N*_*2*_ *vitamin B5*	*1*.*56*	*222*.*1*	*92*.*0*	*10*	*147*.*0*	*12*

Sample preparation. Cell pellets were reconstituted in 20 μL of buffer (pH 7.8), consisting of 100 mM Tris base, 6 M urea, 2 M thiourea and 2% ASB-14 (adjusted to pH 7.8 with HCl). Samples were shaken for 30 minutes (1000 rpm; 37⁰C) before being diluted 1:100 with water and analyzed via UPLC-MS/MS.

### Inductively-coupled-plasma mass spectroscopy (ICP-MS)

Cellular iron content was analyzed by ICP-MS using the protocol previously reported [21]. Briefly, 150 μg of total protein as measured by Bradford protein assay, was lyophilized before resuspension in 100 μl nitric acid (69% v/v; ultraclean grade, Aristar) overnight at room temperature (RT). Samples were then heated for 1 hour at 90°C, before the addition of an equivalent volume of hydrogen peroxide (30%, Merck). Sample was incubated for 15 min at RT before a further 30 min at 70°C. To evaluate metal content against calibration standards (#IMS-102; Ultra Scientific) samples were diluted in double-distilled water until within quantifiable parameters using a NexION 350X inductively coupled plasma mass spectrometer (PerkinElmer, Waltham, MA, USA). Each sample was measured in triplicate and normalized to total protein concentration.

### Live cell imaging

For live cell imaging iPSC cells were incubated with 25 nM TMRM (tetramethylrhodamine, methyl ester; a cell permeant, cationic, red-orange fluorescent dye sensitive probe for mitochondrial membrane potential and used in the redistribution mode) for 40 minutes in a HEPES-buffered salt solution (HBSS) composed of (mM): 156 NaCl, 3 KCl, 2 MgSO_4_, 1.25 KH_2_PO_4_, 2 CaCl_2_, 10 glucose and 10 HEPES; pH 7.35. Images were obtained using a Zeiss 710 Laser Scanning Microscope (CLSM) with an integrated Meta detection system and a 40x oil-immersion objective. Illumination intensity was kept at the minimum of laser output and the pinhole was set to give an optical slice of ∼1 μm. TMRM was excited using the 560 nm laser line and fluorescence measured above 580 nm. Calcein-AM based cell area measurements were used for normalization. The NADH autofluorescence was measured with excitation at 405 nm and emission at 440–480 nm. FAD autofluorescence was determined using 458 nm Argon laser line and fluorescence was measured from 505 to 550 nm.

FAD and NADH redox indexes and mitochondrial pools were estimated by sequentially applying 1 μM of the mitochondrial uncoupler FCCP (carbonyl cyanide p-trifluoromethoxyphenylhydrazone), followed by 1 mM of the complex IV inhibitor sodium cyanide [30]. Lipid peroxidation experiments were performed using, C11-BODIPY (581/591, 2 μM, Molecular Probes) was excited at the 488 and 565 nm laser lines and fluorescence measured from 505 to 550 nm and above 580 nm (40x objective). GSH levels were assessed using monochlorbimane, MCB (Molecular Probes, Invitrogen) fluorescence with excitation at 351 nm and emission at 435–485 nm. Data were acquired and analyzed using ZEN2009 software. Superoxide generation was measured with Dihydroethidium (HEt; 2 μM, Invitrogen). Fluorescent images were acquired with a frame interval of 10 s. Data were analyzed using software from Andor IQ (Belfast, UK). Statistical analysis and data analysis were performed using Origin 9 (Microcal Software Inc., Northampton, MA, USA) software. Results are expressed as means ± standard error of the mean (SEM).

### Sanger sequencing

gDNA was extracted from iPS cells and differentiated neuronal cells from all utilised patients and control clones using a standardized phenol extraction.

Primers and touch-down PCR programmes as available in Table 4 were utilised to Sanger sequence the specified mutations within the PANK2 gene as given in Table 5 for the respective disease and control clones to confirm their presence/absence.

**Table 4:** Primer sequences, amplicon size and TD-PCR programmes for gDNA Sanger sequencing of PANK2-mutations. Detailed Sanger sequencing protocol and cycling conditions upon request.

Patient Number	Mutation	Primer F	Seq	Primer R	Seq	amplicon size	PCR programme
1	R481Lfs*Q	PANK2_x5_F-iPS	tgttgggctttgttgctgtt	PANK2_x5_R-iPS	aacaaaccccaccccaaatg	297	TD65_55
1	G521R	PANK2_x6_F-iPS	catggtgctgtatttggggt	PANK2_x6_R-iPS	ccatttcacctccaccctct	302	TD65_55
2	IVS4–1 G>T = splice acceptor just before exon 5	PANK2_x5spli_F-iPS	tggagggctctgtttgaagt	PANK2_x5spli_R-iPS	tggccaggtcctctttactg	242	TD65_55
2	M437T	PANK2_x4_F	agggaggaggtgttagcatg	PANK2_x4_R	agggaggaggtgttagcatg	458	TD65_55
3	R278L	PANK2_x2_F-iPS	tcggtggaactctggtcaag	PANK2_x2_R-iPS	atttgtacgctccacctcca	309	TD65_55
3	L564P	PANK2_x7_F-iPS	gggtgctcaggaaatgggta	PANK2_x7_R-iPS	aaatcctcacactgcctgga	416	TD65_55

**Table 5:** Overview of clinical and genetic features of atypical PKAN cases. PANK2 transcript: ENST00000316562.

Patient	Mutation	Gender	Age of Onset [y]	Age at biopsy (y)	Current Age [y]	Predominant Phenotype	MRI	FH	Clinical diagnosis
1	R481Lfs*Q + G521R​	M	12	41	Deceased at age 43	Generalised dystonia	Iron deposition on basal ganglia.	Negative	PKAN
2	IVS4–1 G>T = splice acceptor site just before exon 5 + M437T	F	15	39	42	Anarthria, dystonia	Marked hypodensity bilaterally and symmetrically within the globi pallidi.	Negative	PKAN
3	R278L + L564P	M	6	41	45	Generalised dystonia	Eye of the tiger in the globi pallidi.	Negative	PKAN

PKAN—pantothenate kinase associated neurodegeneration. MRI—magnetic resonance imaging, FH—family history, M—male, F—female, y—year.

### Statistical analysis

Data represent three control PSCs and at least two clones from three unrelated PKAN patients. Number of independent inductions is depicted in histograms. Where appropriate, statistical significance was calculated using the Student’s *t* test or ANOVA, as stated. Histograms represent mean values and error bars represent standard error of the mean. *p<0.05, **p<0.01, ***p<0.001.

## Results

### Generation of patient-derived iPSCs and cortical neuronal cells

iPSC lines from three NBIA patients with confirmed *PANK2* mutations were generated to study the mutation effect in human neurons *in vitro* (Table 5). These patients each have compound mutations leading to atypical PKAN with later disease onset and less severe progression than classical PKAN (Table 5). Clinically, these patients display iron accumulation evident by T2* MRI and a generalised dystonia phenotype.

**Fig 1:**
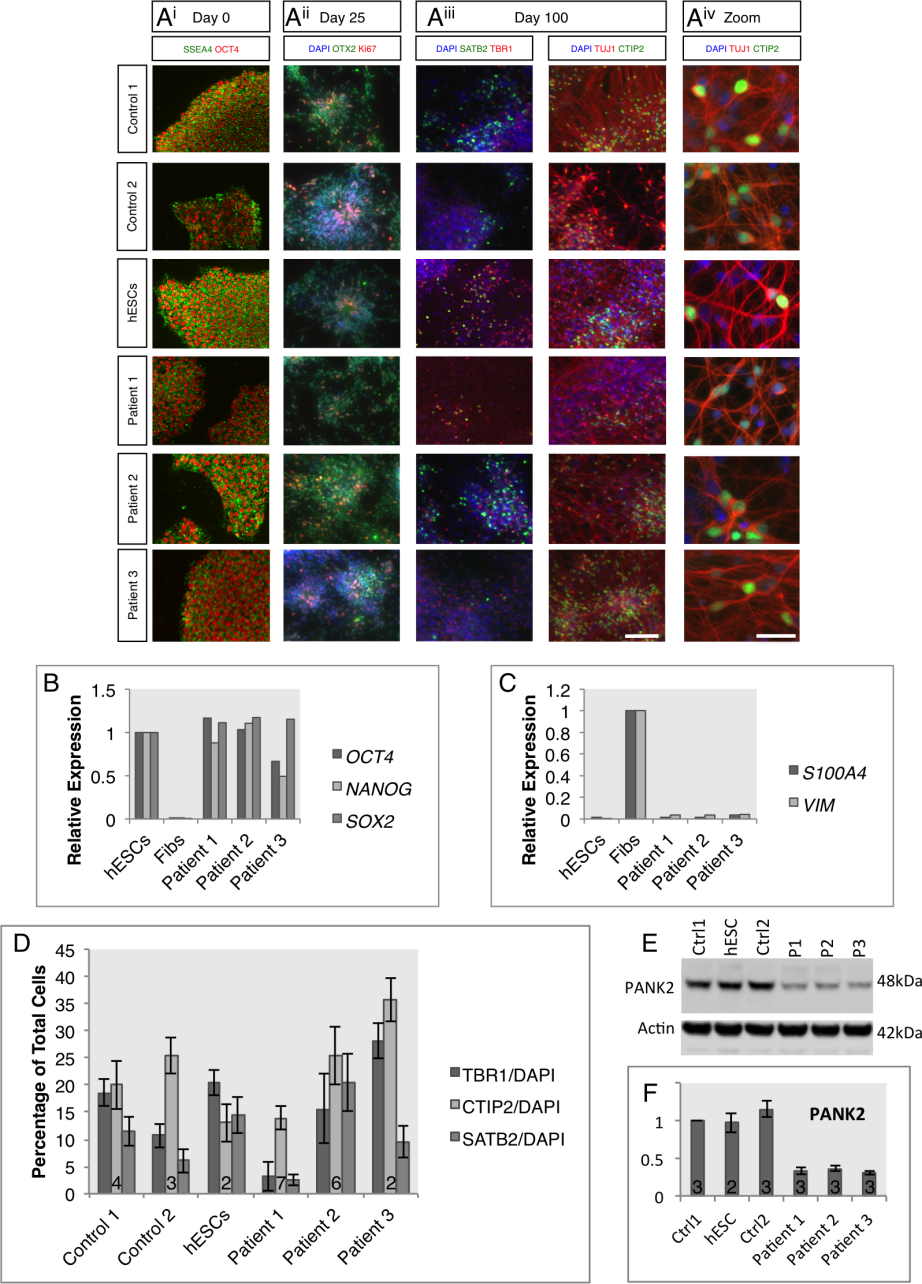
Characterization of *PANK2* iPSCs and differentiated neuronal cultures. A) Immunocytochemistry on three control lines and newly generated iPSC lines from three *PANK2* mutation carriers to confirm pluripotency in iPSC cultures and successful neuronal differentiation. i) Pluripotency of iPSC used in this study was confirmed by positive immunofluorescence for OCT4 and SSEA4. ii) At day 25 of neuronal induction, neuronal precursors were present in all cultures with rosette morphology and showed positive immunofluorescence for the forebrain marker OTX2 and proliferative marker Ki67. iii) After 100 days of differentiation, cultures expressed the pan-neuronal marker β-III-tubulin as well as deep (TBR1-positive), middle (CTIP2-positive) and upper (SATB2-positive) layer cortical markers. iv) Neuron-like morphology was evident at higher magnification. B) Further confirmation of pluripotency in nascent iPSC lines was obtained by qPCR analysis to show expression of the pluripotency markers *OCT4*, *NANOG* and *SOX2*. Expression levels were normalized to those in human embryonic stem cells. Fibroblasts were included as a negative control. C) Downregulation of fibroblast enriched gene expression, *S100A4* and *VIM*, was assessed using qPCR. Normalization to untransfected fibroblasts demonstrated low expression in newly derived iPSCs similar to embryonic stem cells. D) Quantification of the relative proportions of TBR1, CTIP2 and SATB2 positive cells relative to total cell numbers in differentiated cultures. Individual data represent 2 iPSC clones from patient 1, 3 clones from patient 2 and 1 clone from patient 3. E) Representative western blot analysis of PANK2 protein levels in whole cell lysates from control and patient neuronal cultures at day 100 of differentiation. PANK2 was observed at the predicted molecular weight of 48kDa. Actin was used as a loading control. F) Quantification of western blot data from three independent inductions showed consistently lower levels of PANK2 in patient neuronal cells. Scale bar represents 100 μm for all images, and 20 μm for A^iv^. Numbers in histogram bars represent experimental replicates.

Fibroblasts from the 3 patients were reprogrammed to iPSCs using the non-integrating episomal reprogramming method [26]. At least two iPSC clones from each patient were picked and expanded for further characterisation, alongside two control iPSC lines and one control hESC line. To confirm pluripotency in the newly derived iPSC lines, expression of the pluripotency markers OCT4 and SSEA4 were immunocytochemically identified (Fig 1A). *PANK2* iPSCs displayed similar expression of these pluripotency markers and characteristic colony morphology to control lines. qPCR analysis also showed that the gene expression of the core pluripotency markers *OCT4*, *NANOG* and *SOX2* was comparable to hESCs in contrast to nascent fibroblasts (Fig 1B) and that the newly formed iPSCs faithfully silenced fibroblast markers *S100A4* and *VIMENTIN* (Fig 1C). The genomic integrity of the newly formed lines was confirmed by karyotype stability and G-band analysis (S1 Fig) and all disease lines were confirmed to carry the compound heterozygous mutations by Sanger sequencing (S1 Fig), as specified in Table 4. Absence of these mutations was confirmed in control PSC lines.

To generate neurons, we subjected the iPSCs to a cortical differentiation protocol due to the very high efficiency of differentiation (>95%) [27,31]. All lines tested from the patient-derived iPSCs were able to faithfully generate forebrain patterned neural precursor cells, expressing the telencephalic marker OTX2 (Fig 1A). After 100 days of neurogenesis, control and patient-derived PSCs generated deep layer (TBR1-positive), upper layer (SATB2-positive) and middle layer (CTIP2-positive) cortical neuronal cells (Fig 1A). The ability of lines to undergo cortical neurogenesis showed some variability but was comparable between all patient lines (Fig 1D), consistent with a non-developmental disease. Sanger sequencing was performed on genomic DNA from terminally differentiated cells and all patient-derived heterozygous mutations were confirmed (S1 Fig). Western blot analysis demonstrated a reduction of mature PANK2 protein in patient-derived neuronal cultures in comparison to control cell cultures, (patient 1 32.8±4.1%; patient 2 36.5±4.5%; patient 3 30.2±2.1%), despite only one patient displaying a premature stop codon mutation (Table 5).

### CoA homeostasis in patient-derived neuronal cells is comparable to control

Only a subset of mutations in *PANK2* that lead to PKAN reduce enzyme activity and affect protein folding [32,33]. Indeed, changes in CoA levels in human cell lines or brain tissue have yet to be demonstrated [2]. Therefore, absolute levels of CoA and acetyl-CoA were investigated in the cultures via HPLC.

Primary analysis of the free CoA to acetyl-CoA ratio in iPSC cultures and differentiated cells indicated an undefined switch in the metabolic states between the two cell types, seen via a relative decrease in free CoA and an increase in acetyl-CoA levels in neuronal cells. This ratio change between iPSCs and neurons was independent of *PANK2* mutation (S2 Fig).

**Fig 2:**
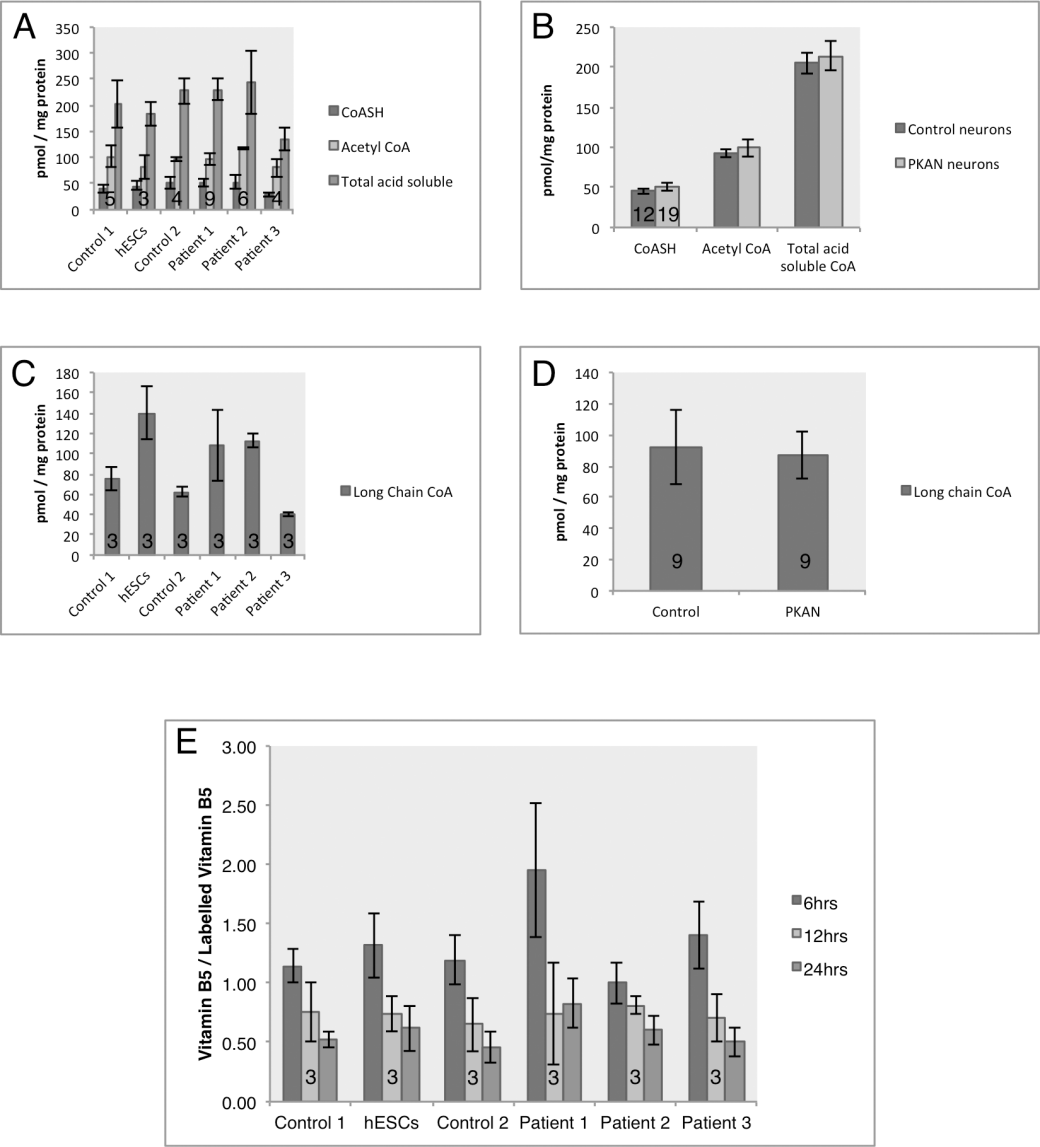
Analysis of the coenzyme A biosynthetic pathway in control and mutant iPSC-derived neurons. A) Analysis of the short chain CoA species by HPLC, with focused analysis on the simple CoA-SH and acetyl-CoA. B) Comparison of pooled data for control and patient-derived neurons displaying comparable levels of short chain acid-soluble CoAs. Patient 1 = 2 clones, patient 2 = 3 clones, patient 3 = 1 clone. C) Analysis of long chain CoA species in the individual lines and D) pooled data, analysed by HPLC. Patient 1 = 1 clone, patient 2 = 2 clones, patient 3 = 1 clone. E) UPLC-MS/MS analysis of uptake of stable isotope vitamin B5 from the media in control and patient-derived neurons, measured as a ratio of total B5 relative to isotopically labelled B5.) Patient 1 = 1 clone, patient 2 = 2 clones, patient 3 = 1 clone. Numbers in histograms represent experimental replicates.

No change in short chain CoA species (free CoA (CoASH), acetyl-CoA and short-chain CoA esters) was observed by HPLC (Fig 2A and 2B). As it has been previously reported that *PANK2* mutations could lead to the disruption of β-oxidation of fatty acids [33], long chain CoA derivatives were also evaluated. While some variability arose between cell lines (Fig 2C), no overall significant difference was observed between control and mutant lines (Fig 2D). Together, these data signify a comparable steady state of total CoA levels in the patient-derived neuronal cells to control cultures.

To infer changes of synthesis and breakdown of CoA in patient-derived neuronal cultures, stable isotope labelled vitamin B5 was used to measure neuronal uptake and handling (Fig 2E). Cells treated with labelled vitamin B5 (pantothenate) for up to 24 hours displayed a similar rate of uptake between control and patient-derived neuronal cultures (Fig 2E). No change also inferred that the cellular lifetime of vitamin B5 was unaffected by the presence of the *PANK2* mutation, shown via similar homeostasis of the labelled pantothenate.

### Patient-derived neuronal cells exhibit dysfunctional mitochondrial respiration

In order to test whether the mutations in *PANK2* affect the mitochondrial health in our iPSC-derived neuronal cells, mitochondrial membrane integrity and live cell imaging experiments were performed.

**Fig 3:**
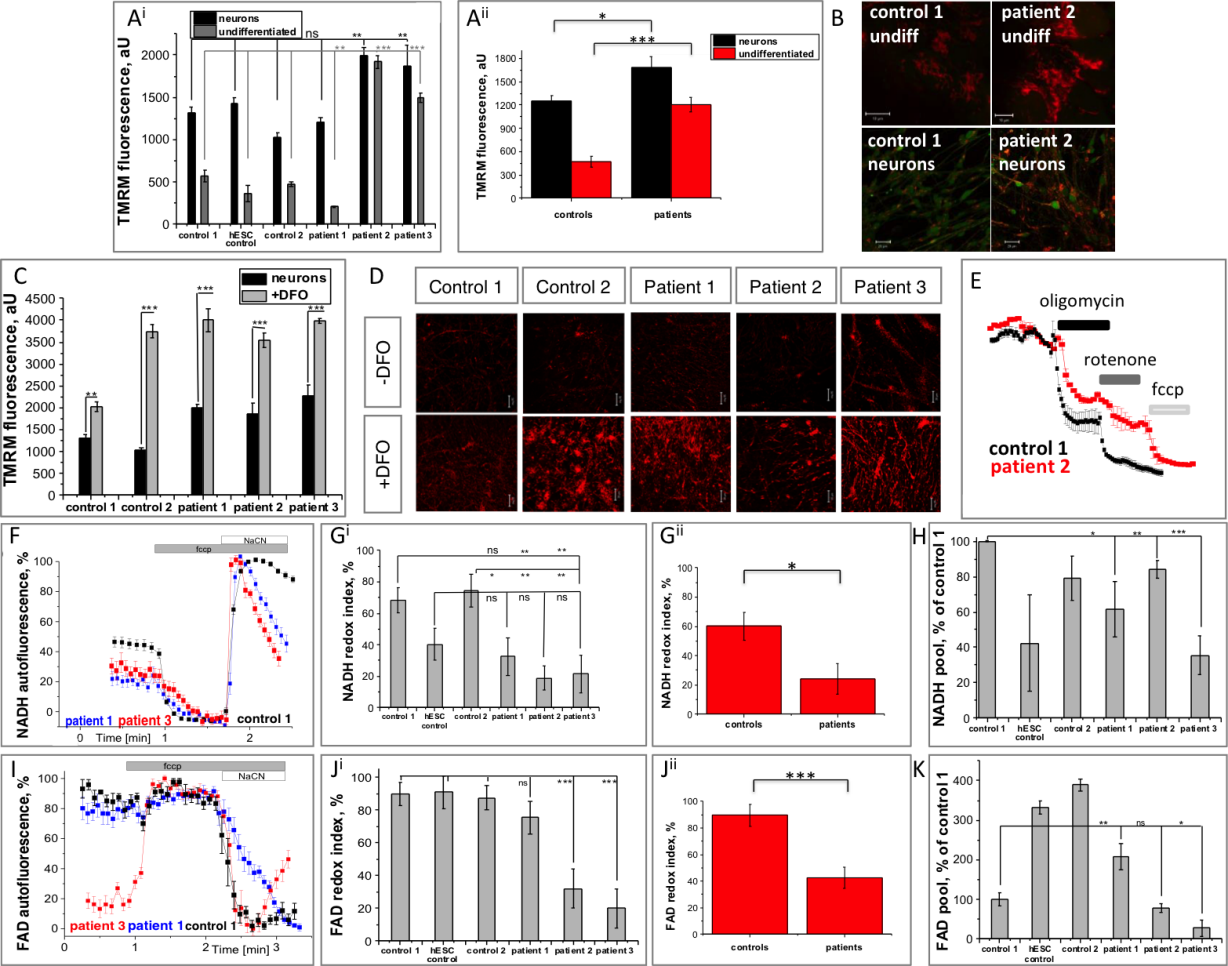
Bioenergetic profile of PANK2-deficient patient-derived neurons. A) Analysis of mitochondrial membrane potential using the fluorescent dye TMRM in stem cells and neurons from control and patient-derived lines. Data from individual lines (A^i^) and pooled data of controls versus patients (A^ii^) is presented. B) Representative images of TMRM fluorescence (red) and calcein (to depict cell borders–green). C-D) Neurons were pre-treated for 30 minutes with the iron chelator DFO prior to TMRM measurement. Pre-treatement led to an increase in mitochondrial membrane potential in all lines. E) Representative traces of analyses into electron transport chain function by sequential application of inhibitors to complex V (oligomycin), complex I (rotenone) and the mitochondrial uncoupler (FCCP). Rotenone caused a partial depolarisation in patient cells, demonstrating dysfunction of complex I. Oligomycin caused a substantial depolarization in both control and patient-derived cells. F-H) Analysis of NADH autofluorescence to quantify the redox index of NAD^+^/NADH (G^i^ individual lines and G^ii^ pooled data) revealing reduced redox index in patient derived cells. H) Quantification of the total NADH pool. I-K) Analysis of cellular FAD reveals a reduced redox index in patient-derived cells (J^i^ and J^ii^) despite similar mitochondrial pools (K). Scale bar represents 10 μm or 20 μm as depicted. Significance was calculated via student’s t test (C, H, K) and one way ANOVA with post-hoc Tukey’s HSD correction for multiple comparisons (A, G, J) *p<0.05, **p<0.01, ***p<0.001, ns, not significant.

Mitochondrial membrane potential (Δψ_m_), is an indicator of mitochondrial function, and was assessed using TMRM fluorescence (Fig 3A and 3B). The presence of the *PANK2* mutation increased Δψ_m_ in both iPSC (pooled controls: 470.3±72.2, n = 48 versus pooled patients: 1207.0±95.3, n = 72. ***p<0.0001) and neuronal cells (pooled controls: 1254.3±67.7, n = 48; versus pooled patients: 1685.6±135.1, n = 106; p = 0.005, Fig 3A^ii^). Changes in mitochondrial membrane potential have been reported in other PKAN models [7,16], albeit with opposing results. Here, altered maintenance of Δψ_m_ is likely to represent a compensatory mechanism to mitochondrial deficits. Some variability between cell lines was apparent, as demonstrated by patient 2 and 3 derived cells displaying significantly higher Δψ_m_ compared with controls, whereas patient 1 derived cells consistently demonstrated Δψ_m_ similar to control lines (Fig 3A). Due to this variability, data for individual cell lines is presented, and significant comparisons are depicted by the pooled data (Fig 3A^ii^).

The known association of PKAN pathology with brain iron accumulation led us to examine whether iron was involved in changes to the mitochondrial membrane potential observed in patient cells (Fig 3C and 3D). Pre-treatment with the iron chelator deferoxamine (DFO) at 50 μM for 30 minutes increased neuronal Δψ_m_ (control 1: 1317.7±65.8 to 2023.8±104.3, n = 48; control 2: 1025.7±62.8 to 3741.2±149.3, n = 49; patient 1: 1987.3±96.2 to 3999.4±270.7, n = 33; patient 2: 1864.0±256.1 to 2272.3±238.3, n = 49; patient 3: 2272.3±238.3 to 3989.1±51.6, n = 49. ***p<0.001) representing a further increase in membrane potential above non-treated PKAN-associated levels as a result of iron chelation (Fig 3C and 3D).

The basis for differences in Δψ_m_ maintenance was further investigated by exposing the cells to known specific inhibitors of the electron transport chain complexes (Fig 3E). A comparable decrease (~70% of basal levels) in control and PKAN neuronal cells of TMRM signal after oligomycin treatment (complex V inhibitor), suggested complex V in these cells was working in reverse as an ATPase to maintain mitochondrial membrane potential. Rotenone (a complex I inhibitor) also induced a decrease of TMRM fluorescence, however PKAN cells responded slower and to a reduced extent than control cells (Fig 3E). This suggested that either complex I in PKAN neuronal cells was ineffective at generating a membrane potential or NADH substrate availability from the TCA cycle was decreased.

To elucidate whether complex I was dysfunctional or starved of substrates, mitochondrial NADH redox levels were measured (Fig 3F and 3G) and found to be significantly reduced in PKAN neuronal cells under basal conditions (pooled controls: 60.1±9.5%, n = 24; versus pooled patients: 24.3±10.6%, n = 36. *p = 0.0139). The application of the mitochondrial uncoupler FCCP, to fully deplete NADH levels by stimulating mitochondrial respiration, and NaCN, to block mitochondrial respiration and thus NADH consumption, enabled the rate of NADH production and maximal pool of NADH in mitochondria to be estimated (Fig 3F–3H). A trend towards a reduced total pool of NADH was evidenced in PKAN neuronal cells. Together these observations were thus able to explain the reduction in complex I activity due to lack of NADH substrate availability.

Similar analysis of the complex II substrate FAD revealed that PKAN neuronal cells displayed a significantly lower FAD redox index (pooled controls: 89.5±8.2%, n = 24; versus pooled patients; 42.4±8.0%, n = 36. ***p = 0.0001) (Fig 3I–3K), indicating an inhibited rate of complex II dependent respiration. Altogether, these investigations provide evidence for a defective electron transport chain in neurons carrying the *PANK2* mutation.

### Increased ROS production and oxidative stress in PKAN patient-derived neuronal cells

**Fig 4:**
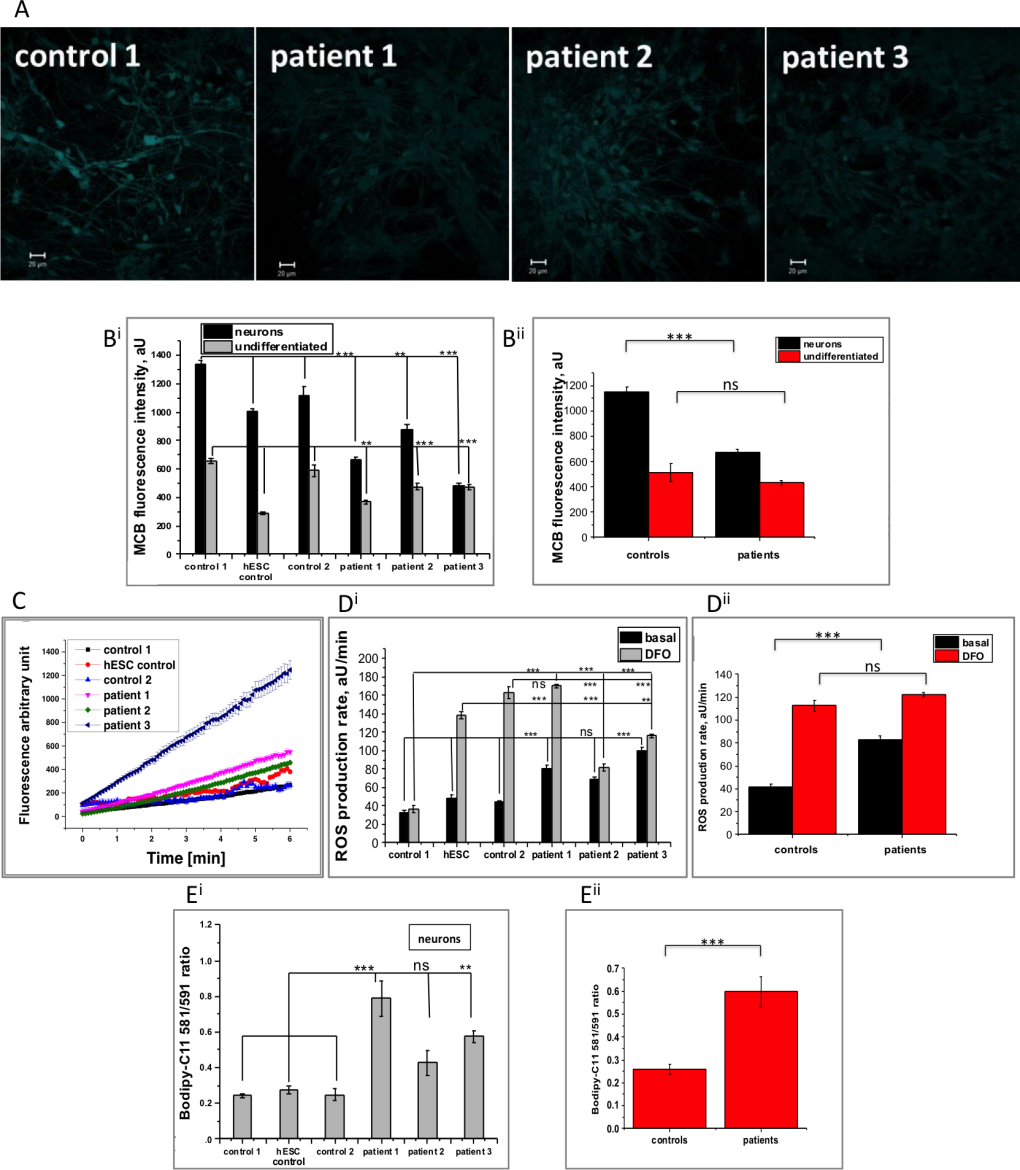
*PANK2* mutations result in increased rate of ROS production, depletion of cellular GSH and higher oxidative stress. A) Representative images of glutathione levels in control and patient-derived neurons measured by monochlorobimane (MCB) fluorescence in live cells. B) Quantification of MCB fluorescence in iPSCs and neuronal cells showed reduced glutathione levels in patient-derived cells and lower levels in iPSCs, (B^ii^ shows pooled data). C) Basal rates of ROS production were quantified in neurons using the dihydroethidium assay. D) Quantification showed an increased rate of ROS production in patient cells compared to controls and in all cell lines in response to 30 minutes pre-incubation with iron chelator DFO (D^ii^ depicts pooled data from D^i^). E) The level of lipid peroxidation was quantified in the neuronal cells using the ratiometric dye BODIPY C11 and two of the patient-derived neuronal cells displayed significantly higher levels of lipid peroxidation (pooled data shown in E^ii^). Scale bar represents 20 μm. Significance was calculated via one way ANOVA with post-hoc Tukey’s HSD correction for multiple comparisons *p<0.05, **p<0.01, ***p<0.001, ns not significant.

Using the fluorescent dye monochlorobimane (MCB), levels of the reduced form of the antioxidant glutathione (GSH) were measured in control and patient-derived neuronal cells (Fig 4A and 4B). Compared with controls cells, MCB fluorescence intensity was significantly decreased in both undifferentiated patient-derived iPSCs (pooled controls: 512.3±73.5, n = 90; versus pooled patients: 431.0±17.6, n = 127. p = 0.3022) and PKAN neuronal cells (pooled controls: 1151.0±36.6, n = 105; versus pooled patients: 675.6±21.7, n = 120. ***p<0.0001), representing a decreased intracellular antioxidant pool. The lower reduced glutathione level in iPSCs compared to neuronal cells (Fig 4B) is consistent with the low oxidative phosphorylation status and ROS production of iPSCs that metabolically correspond to an early embryo [34].

ROS generation rates in the neuronal cells were measured by dihydroethidium oxidation as a direct readout of superoxide (O_2_^-^) production. Under basal conditions, O_2_^-^ generation was consistently higher in PKAN neuronal cells compared with control cells (pooled controls: 41.7±2.6, n = 46; versus pooled patients: 83.2±3.0, n = 54. ***p<0.0001) (Fig 4C and 4D). Lipid peroxidation, assessed via the ratiometric dye BODIPY-C11, was also found to be significantly higher in PKAN neuronal cells from two of the patients whereas cells derived from patient 2 showed a similar trend but did not reach significance (0.59683±0.06744, n = 196 for pooled patients, compared to 0.26±0.022, n = 86 for pooled controls; ***p<0.0001 Fig 4E).

Following the findings in Fig 3C, the relationship with iron and PKAN neuron associated oxidative damage was explored. Surprisingly, 30 minutes pre-treatment of neuronal cells with DFO increased ROS generation in both control and patient-derived cells (pooled controls: 112.5±4.6; n = 46; versus pooled patients: 122.0±2.4; n = 53. p = 0.0782) (Fig 4D).

Taken together, these data provide evidence that the mitochondrial dysfunction observed in patient-derived neuronal cells leads to increased ROS generation and downstream oxidative damage. In our conditions, iron chelation exacerbated ROS generation and worsened the PKAN neuronal phenotype.

### Cellular response to iron in PKAN patient-derived neuronal cells

Iron accumulation is a progressive pathological characteristic of PKAN seen via MRI during life [35], however, it is unclear whether this is a cause of neurodegeneration or a consequence. Here, the iron response pathway was assessed in control and patient-derived neuronal cells.

**Fig 5:**
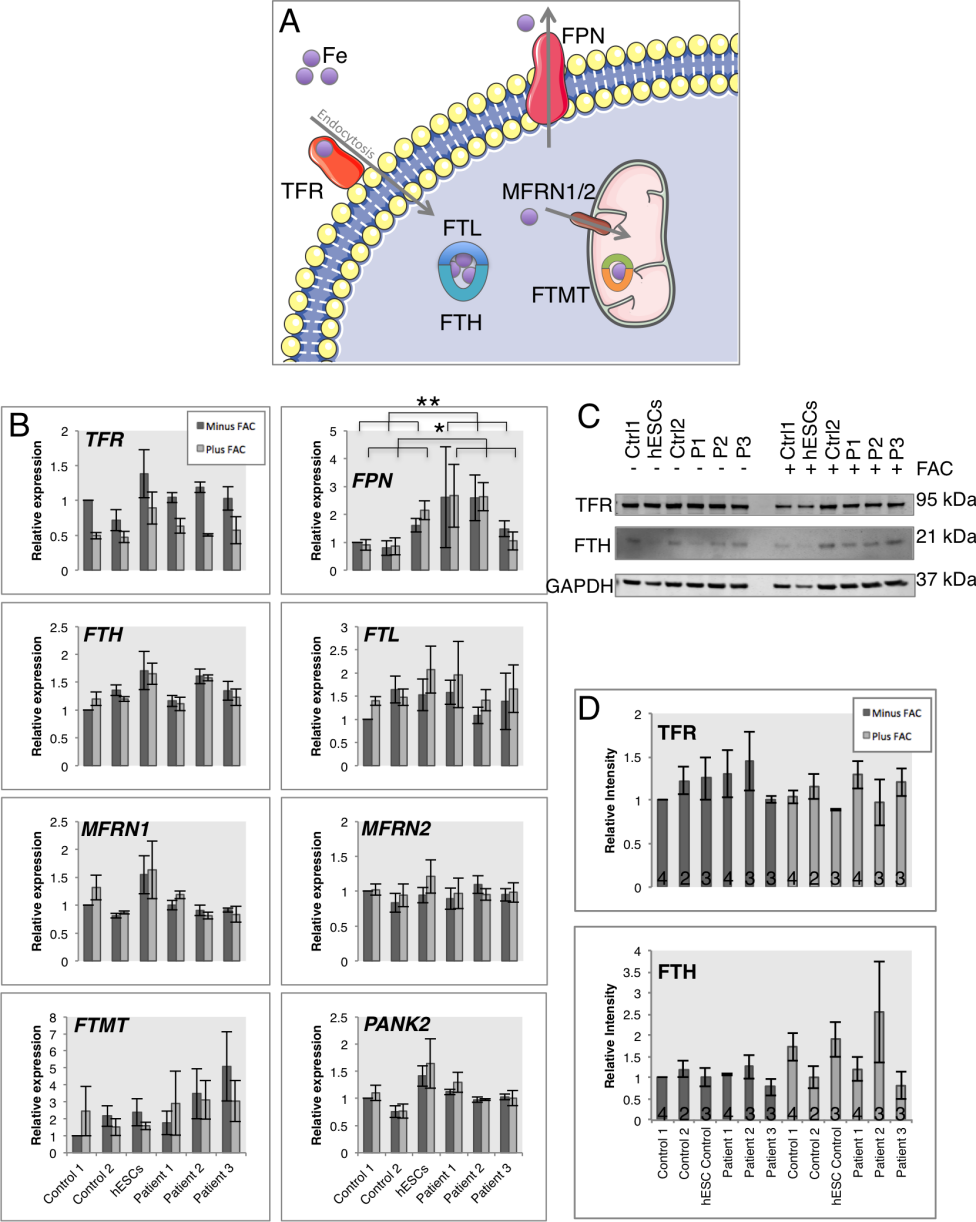
Neuronal response to excess extracellular iron treatment. A) Schematic representation of the iron handling gene products analyzed in this figure. Transferrin receptor (TFR) binds Transferrin bound extracellular iron and is internalised via endocytosis. Ferritin molecules FTH and FTL bind the cytosolic labile iron pool. Iron import to mitochondria is performed by Mitoferrins (MFRN1 and MFRN2) and excess mitochondrial iron is stored in Mitochondrial Ferritin (FTMT). Neuronal iron export is performed by Ferroportin (FPN). B) Quantitative PCR analysis of iron responsive genes under basal conditions (dark grey bars) and after 18 hours of 50 μM ferric ammonium citrate treatment (light grey bars). C) Western blot analysis of iron responsive proteins TFR and FTH. D) Densitometry analysis of the western blots in C). Patient 1 = 2 clones, patient 2 = 2 clones, patient 3 = 2 clones. C-D) Patient 1 = 1 clone, patient 2 = 1 clone, patient 3 = 1 clone. *p<0.05, **p<0.01. Numbers in histograms represent experimental replicates, and n = 4 throughout B).

Response of the cellular iron-handling pathway was analyzed in response to 18 hours iron treatment (50 μM ferric ammonium citrate (FAC)) (Fig 5A). Across all cell lines and consistent with a reduction in cellular iron import, TfR expression was reduced at the transcriptional and expression levels (Fig 5B–5D). The cytosolic iron storage molecule Ferritin (FTH and FTL) exhibited increased protein levels in all lines, consistent with its translational control by iron response genes [36].

Interestingly, *Ferroportin* expression was significantly increased in patient-derived neuronal cells versus controls, in basal conditions and in response to iron treatment (Fig 5B), suggesting a cellular response for increased iron export. No significant transcriptional change in response to iron was observed for *MFRN1/2 or PANK2*, however, a trend to increased *FTMT* expression was witnessed in patient-derived cells, hinting to increased mitochondrial iron storage (Fig 5B). It is noteworthy that *PANK2* expression is similar between control and patient-derived neuronal cells, whereas protein levels are reduced in patient-derived cells (Fig 1E and 1F).

### Total iron content is unchanged in patient-derived neuronal cultures

**Fig 6:**
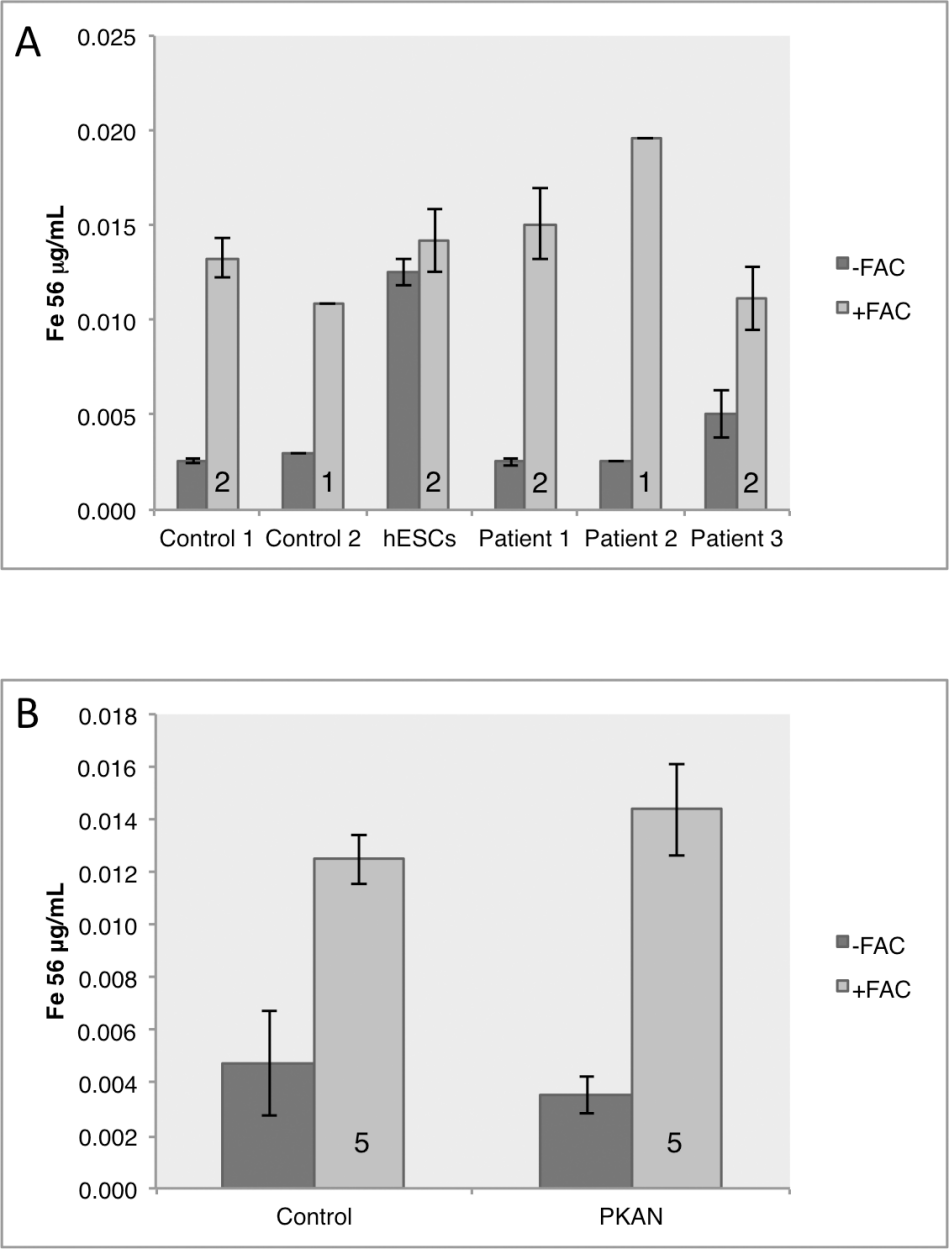
Analysis of the neuronal iron content. Neuronal pellets were analyzed for total iron content by ICP-MS in basal conditions and after 16 hours of FAC media supplementation. A) Individual data shown for each biological replicate and B) shows mean results for control lines versus patient-derived neuronal pellets. Control and patient-derived neurons displayed similar levels of basal iron content as well as similar storage of excess iron after iron stress. Numbers in histograms represent experimental replicates.

To investigate whether the small differences in expression of *FTMT* and *FPN* in response to extracellular iron (Fig 5B) related to altered cellular iron content, absolute metal ion content was measured by inductively coupled plasma mass spectrometry (ICP-MS). In all neuronal lines, elevated intracellular iron content upon incubation with FAC (50 μM; 18 hours) was confirmed and no change was observed between control and PKAN neuronal cells (Fig 6).

Data indicates a largely appropriate iron response from PKAN neuronal cells that preserves intracellular levels of iron even in an elevated extracellular iron environment. An exception is the consistent increase in *FPN* expression in patient-derived cells versus controls in both basal conditions and under iron stress, which may indicate a compensatory measure to maintain intracellular iron levels by enhancing iron export.

## Discussion

In this study, PKAN patient-derived neuronal cells have been generated in an attempt to identify early mechanisms of neurodegeneration in NBIA. These cells represent a human model with appropriate gene dosage of clinically proven pathogenic mutations in which to study the earliest underlying consequences of *PANK2* mutations in developmentally immature neuronal cells.

Cortical neurons represent a relevant model of PKAN, as one of the cell types affected by axonal swellings and gliosis, additional to the main site of pathology in the globus pallidus [19]. There currently exists no stem cell differentiation protocol towards pallidal neurons and the cortical differentiation protocol employed here is highly efficient and generates very homogenous cortical cultures due to the default nature of this developmental paradigm [27]. It should be noted that other cell types exist in the culture, for example glial cells and some progenitor cells that persist, together representing less than 5% of cells [27]. These cells represent a minority, however we cannot discount contributions of these cells to our data, for example the provision of metabolites from astrocytes to neurons for oxidative phosphorylation.

We observed a significant degree of variability in cellular responses to many experimental paradigms; including the fact that cells derived from patient 1 often responded similar to controls. These variabilities may represent tissue culture artefact and a consequence of the cells being cultured outside of their natural context and one example of this is the substantial change in transcription witnessed in primary microglia just six hours after being placed in culture [37].

Our observation that the relative proportion of free CoA to acetyl-CoA changes through differentiation suggests a metabolic alteration between the stem cells and neurons. This finding calls for future investigations to describe the metabolic states of the two cell types and changes through neuronal differentiation.

We find that point mutations associated with atypical PKAN lead to unaltered *PANK2* expression in neuronal cultures but a reduction in PANK2 protein levels. Hypothetically, this could be explained via alterations in folding and degradation of the putative unfolded protein; further investigation is required. These findings are in line with iPSC-derived neurons harbouring premature stop codons; showing unaltered *PANK2* transcription but a total lack of protein [17]. It is noteworthy that biochemically, a number of peptides harbouring PANK2 mutations display normal enzyme function [32]. Thus protein dosage may be central to the disease mechanism, with juvenile onset displaying no PANK2 protein and atypical PKAN a reduced level.

The data presented here demonstrates that pathogenic point mutations in *PANK2* do not alter neuronal CoA levels or the metabolic flux of pantothenate. This suggests that there is either a distinct cellular function for mitochondrial PANK2 or that cytosolic PANK enzymes may be compensating for a PANK2 deficiency. The ability of mitochondrial human PANK2 and only mitochondrial isoforms of drosophila Pank to rescue phenotypes in Drosophila Pank knockouts [13], supports the former concept, whereas the ability of human PANK3 and PANK4 to partially rescue knockout flies supports the latter. Alternatively, CoA flux might respond in a faulty manner in relation to stress in the setting of the ageing or diseased brain.

Dysfunctional mitochondrial oxidative phosphorylation may be a key component in the PKAN brain and altered mitochondrial membrane potential has been seen in other PKAN models. Reduced mitochondrial membrane potential and defective ATP production has been described in PANK2 knockout mouse models, patient-derived fibroblasts as wells as induced neuronal models [7,16]. The increased membrane potential seen in this study may represent a compensatory response of the cells to mitochondrial deficits, in the context of atypical disease mutations. Mitochondrial and metabolic deficiencies could explain the adult onset of neurodegeneration through altered environmental stresses and diet in this highly energy demanding cell type. This is reinforced by mutations in *PANK1* being linked with hyperglycaemia [38,39]. We report a metabolically immature neuronal phenotype in PANK2 mutants as well as in control lines, as seen by depolarization of mitochondria in response to oligomycin application after 100 days of differentiation. This result highlights that all cultures analysed in this study are partially glycolytic, contrary to mature primary neurons in culture. Due to this observation, some metabolic consequences of *PANK2* mutations may be masked by the fact that our cultures are not entirely dependent on mitochondrial oxidative phosphorylation.

Closer analysis of the mitochondrial physiology demonstrated that the cells were defective in complex I of the electron transport chain. Our data indicate that a lack of NADH substrate provided by the TCA cycle may be central to this deficiency. In addition to NADH, *PANK2* mutations reduce the amount of FADH production in complex II. This observation is reinforced by changes in NADH in tissue homogenate in *Pank1*/*Pank2* double knockout mice [11] and validates the hypotheses gleaned in studies in iPSC-derived neurons [17]. Importantly, irrespective of *PANK2* mutations, our cultures rely on glycolysis to compensate for inefficient oxidative phosphorylation and for increased ATP consumption by the ATPase to maintain the mitochondrial membrane potential. It is interesting that undifferentiated iPSCs display some mitochondrial abnormalities in addition to neuronal cells, namely increased TMRM fluorescence and reduced antioxidant levels. Levels of antioxidants have been shown to be altered in PKAN patient-derived fibroblasts and transdifferentiated neurons, including lower levels of reduced glutathione [15,16]. However, in the brain an increase in levels of glutathione-cysteine have been described, suggesting increased oxidised glutathione and potentially oxidative stress [40]. These consistencies again suggest a selective vulnerability of certain neurons to a consistent mutation-associated phenotype.

The reported ultrastructural disruption of cristae organization and mitochondrial swelling in a PKAN transgenic model [14] and in iPSC-derived neurons [17] could occur as a cause or result of the metabolic dysfunction described here. This physical disruption of the respiratory chain could lead to electron leakage and could be another reason for the reversal of the ATP synthase to maintain mitochondrial membrane potential described here. ROS is a normal cellular signal for multiple physiological processes; however prolonged exposure to high levels combined with environmental stresses will inevitably lead to damaged cellular components. Increased ROS generation and subsequent lipid peroxidation as a result of altered oxidative phosphorylation in PKAN neurons could potentially explain the post-developmental onset of the NBIA.

Of note, is the finding that iron chelation increases mitochondrial membrane potential and ROS generation in both control and mutant cells. This can be explained as DFO is a specific Fe(III) chelator, altering the equilibrium between the two redox states. In turn, DFO thus favours the oxidation of Fe(II) to Fe(III), leading to the release of electrons and the formation of ROS. This is an important finding with respect to the relevance of iron to mitochondrial homeostasis, however, it is important to consider that we do not see iron accumulation in our model. For this reason, we cannot comment on the effectiveness of iron chelation with respect to ongoing clinical trials in PKAN using the cell permeable iron chelator deferiprone [4]. Current findings calls for further investigations in other model systems that display iron accumulation and elucidating the role of iron in healthy mitochondria.

Iron deposition is a characteristic feature of NBIAs such as PKAN and is increasingly apparent as an early pathological feature in other neurodegenerative diseases such as AD and PD [41]. It is still unclear whether defective iron homeostasis is a cause or consequence of the neuropathological events in these diseases, but brain imaging has identified that its accumulation is clearly progressive during life [42] and may occur prior to symptom onset [43]. The homeostatic response of iron regulatory proteins and total intracellular iron levels appear largely normal in patient-derived neuronal cells under these conditions. However, altered *FPN* expression reported here and in *PANK2* knockout cell lines [44] suggest an increase in the iron export pathway may exist. A trend to increased *MTFT* expression in *PANK2* mutant neuronal cultures not only reinforces a mitochondrial defect, but also may indicate a further attempt by the cell to sequester excess iron safely. It is tempting to speculate that a mitochondrial defect could lead to altered iron storage in the mitochondrial matrix and increased iron export via FPN, leading to iron dyshomeostasis and a potential accumulation over time. Thus, mitochondrial phenotypes may theoretically precede iron accumulation and underlie PKAN disease progression.

Orellana et. al. performed investigations into PKAN in cortical neurons derived from iPSCs. This study focused on early onset-associated mutations that lead to a lack of mature PANK2 protein. The authors also see mitochondrial abnormalities and increased ROS production in iPSC-derived cortical neuronal cultures, albeit it with reduced mitochondrial membrane potential. This may hint to different compensatory mechanisms between early-onset and late-onset-associated mutations in PANK2 [17]. The authors also hypothesise altered NADH supply to the mitochondria; here we have shown this link to be valid via reduced NADH redox ratios. Orellana et al elegantly show that the wild-type PANK2 transgene can reverse the disease phenotypes, but also put forward extracellular CoA supplementation as a novel therapeutic avenue [17,45]. This strategy has also been shown to reverse disease phenotypes in model organisms of CoA imbalance, as developmental, vasculature and metabolic deficiencies from Coasy and Pank2 knockdown zebrafish are also reversed via CoA supplementation [46,47].

In conclusion, the generation and characterisation of PKAN patient-derived iPSC neuronal cells has provided new insights into the underlying mechanisms of NBIA with relevance to other diseases exhibiting iron accumulation. Reduced cofactor supply for oxidative phosphorylation can explain the mitochondrial defects in patient-derived neuronal cultures, which in turn precedes iron accumulation. Additionally, the effects of iron chelation described here call for careful consideration in the future therapeutic strategies.

## Supporting information

S1 Fig Characterisation of patient-derived iPSC lines. Karyographs to show karyotype stability in the patient-derived reprogrammed iPSCs. All patient-derived iPSCs displayed normal karyotype and g-banding. Confirmation of heterozygous mutations in differentiated iPSC-derived neurons (at least 100 days), mutations depicted within red boxes. gDNA–genomic DNA.
(TIFF)

S2 Fig HPLC analysis of the CoA to acetyl-CoA ratio, comparing iPSCs and neurons. This change in ratio represents a change in respiration dependency on glycolysis and oxidative phosphorylation. Numbers in histograms represent experimental replicates.
(TIFF)
