# Supplementary figures and images for: iPSC-derived neuronal models of PANK2-associated neurodegeneration reveal mitochondrial dysfunction contributing to early disease
Source: PLoS One. 2017 Sep 1;12(9):e0184104. doi: 10.1371/journal.pone.0184104 (PMC5581181; doi:10.1371/journal.pone.0184104)

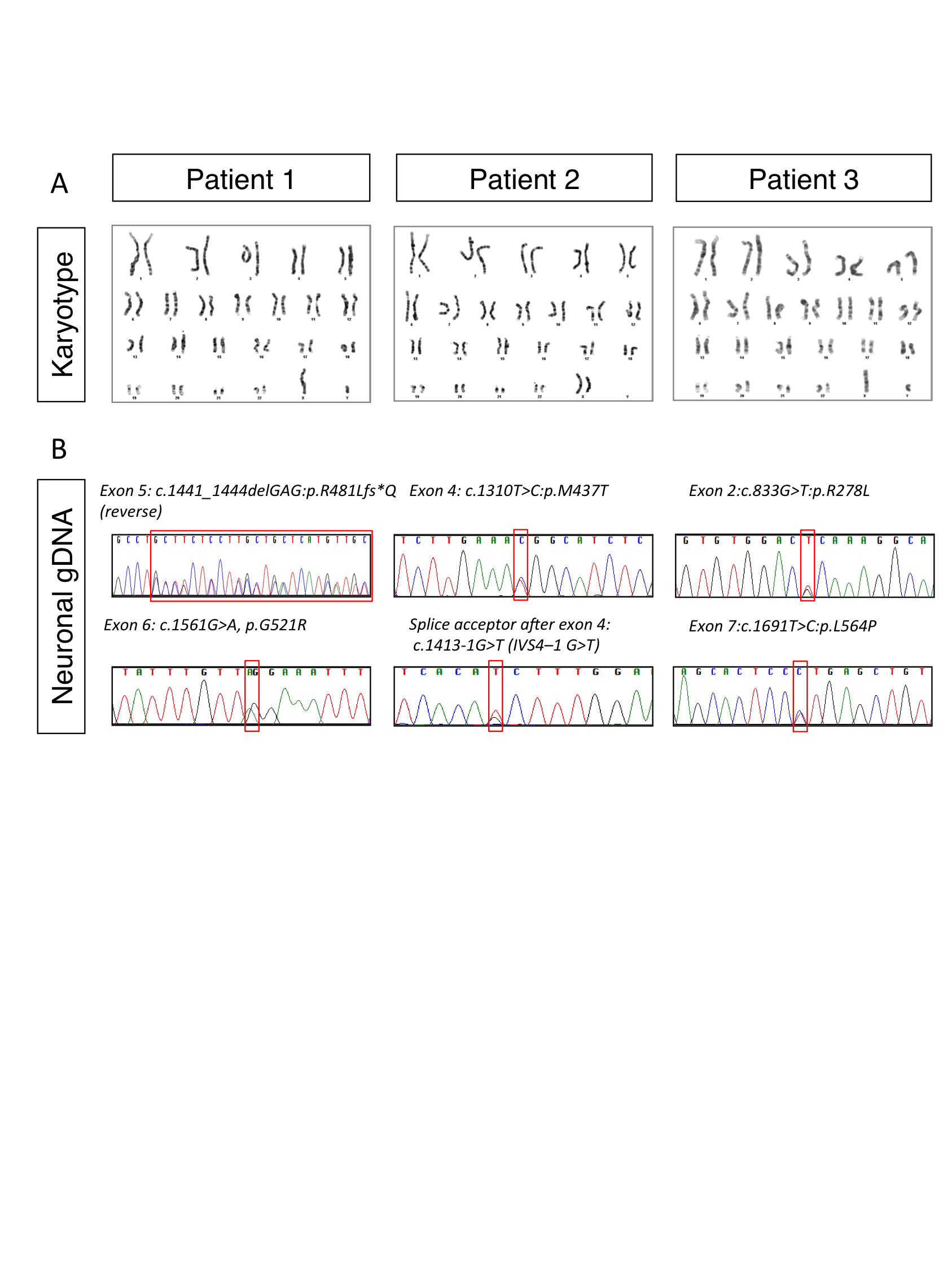

Supplement: S1 Fig — Karyographs to show karyotype stability in the patient-derived reprogrammed iPSCs. All patient-derived iPSCs displayed normal karyotype and g-banding. Confirmation of heterozygous mutations in differentiated iPSC-derived neurons (at least 100 days), mutations depicted within red boxes. gDNA–genomic DNA. (TIFF) [file pone.0184104.s001.tiff]

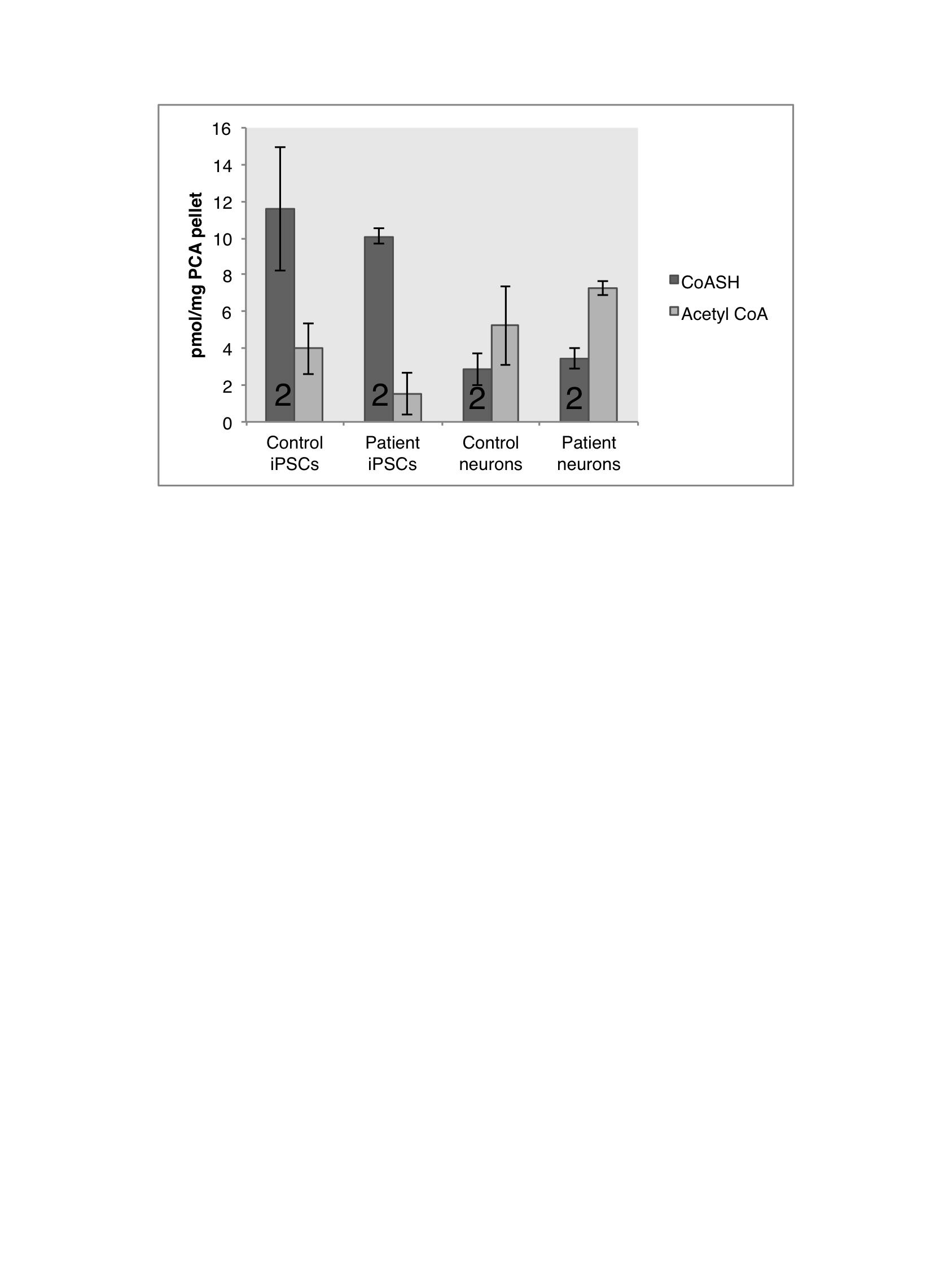

Supplement: S2 Fig — This change in ratio represents a change in respiration dependency on glycolysis and oxidative phosphorylation. Numbers in histograms represent experimental replicates. (TIFF) [file pone.0184104.s002.tiff]
